# Design of Dual Continuous-Mode Class-J Power Amplifiers with Harmonic Matching Networks for X and Ku Bands

**DOI:** 10.3390/mi16121362

**Published:** 2025-11-29

**Authors:** Yang Yuan, Xuesong Zhao, Jingxin Fan, Zhongjun Yu

**Affiliations:** 1 Aerospace Information Research Institute, Chinese Academy of Sciences, Beijing 100190, China; 2 School of Electronic, Electrical and Communication Engineering, University of Chinese Academy of Sciences, Beijing 101408, China

**Keywords:** Class-J, continuous mode, harmonic control, load-pull, MMIC, power amplifier

## Abstract

In this article, two wideband high-efficiency Class-J power amplifiers operating in X and Ku bands, respectively, are designed based on continuous mode. The optimal impedance regions of the transistors are determined using harmonic load-pull techniques. An on-chip output matching network with second harmonic control functionality is designed to achieve Class-J operation. To verify the feasibility of designed circuits, both power amplifiers are designed and fabricated using a 0.25 mm GaAs pseudomorphic high electron mobility transistor (pHEMT) process. The power amplifiers are both biased at 6 V/−1 V. The measured results show the X-band and Ku-band power amplifiers achieve peak saturated output powers of 31.2 dBm and 30.8 dBm, respectively. The power-added efficiencies (PAEs) of the two amplifiers within their operating bands reach up to 48% and 45.3%, respectively. Compact size and high efficiency make them suitable for integration into phased array transmit/receiver (T/R) modules.

## 1. Introduction

Modern active phased arrays demand multifunctionality and lightweight design [1]. Accordingly, the widebanding and miniaturization of transmit/receiver (T/R) modules become imperative. Among these requirements, thermal design is a core factor restricting the miniaturization of T/R modules, while the efficiency of T/R modules is primarily determined by their internal power amplifiers [2]. However, the widebanding of power amplifiers leads to efficiency degradation. Therefore, designing wideband high-efficiency power amplifiers is key to realizing the multifunctionality and lightweight design of phased arrays.

Based on waveform engineering analysis, continuous-mode power amplifiers maintain high efficiency over a wide bandwidth, making them ideal for designing saturated power amplifiers in T/R modules [3]. A waveform-engineered sequential load-modulated balanced amplifier, which employs continuous Class-F^−1^ and Class-J operations, achieves a saturated drain efficiency of 60.2–68.3% across 1.80–2.75 GHz [4]. An ultrawideband power amplifier utilizing a novel multi-branch matching network and extended continuous Class-B/J modes delivers a drain efficiency of 56–70% over 0.4–3.8 GHz, with a relative bandwidth of 162% [5]. The power amplifiers reported in [4,5] employ off-chip high-Q output matching networks (OMNs), achieving high efficiency over a wide bandwidth. However, their large overall footprint renders them incompatible with the integration into transmit/receive (T/R) modules. In [6], an X-band Class-J power amplifier based on active load modulation is implemented in a 0.25 mm GaAs pseudomorphic high electron mobility transistor (pHEMT) process, achieving a peak power-added efficiency (PAE) of 50% over 9.1~10.8 GHz with a die area of 1.89 × 1.69 mm^2^. Ref. [7] demonstrates a broadband power amplifier fabricated in a 0.25 μm GaN HEMT process, realizing a PAE of 36.3–47% across 2–6 GHz.

In this article, two wideband high-efficiency Class-J power amplifiers operating in the X band and Ku band, respectively, are designed and fabricated using a 0.25 mm GaAs pHEMT process. On-chip second harmonic control network is integrated into the OMN to realize the Class-J operation. The measured results show that both power amplifiers exhibit high PAE within their respective bandwidths. Furthermore, their compact footprint renders them suitable for integration with miniaturized T/R modules.

## 2. Continuous-Mode Class-J Power Amplifier

Continuous-mode power amplifiers expand the solution space for the optimal load impedance, thereby enhancing the design flexibility of the output matching network and allowing the power amplifier to maintain high drain efficiency over a broad frequency band [8].

Taking the continuous Class-J power amplifier as an example, the drain-source current *i_d_J_* and voltage *v_d_J_* of the transistor can be given by Equations (1) and (2), respectively [9].

(1) $$i_{d\_J}=\frac{I_{m}}{\pi }+\frac{I_{m}}{2}\cos\theta +\frac{2I_{m}}{3\pi }\cos2\theta -\frac{2I_{m}}{15\pi }\cos4\theta +\ldots$$

(2) $$\begin{array}{l} v_{d\_J}=V_{D}\left(1-\cos\theta \right)\left(1-\alpha \sin\theta \right)\\ \;\;\;\;\;\;=V_{D}\left[1-\sqrt{\alpha^{2}+1}\sin\left(\theta +\arctan\frac{1}{\alpha }\right)+\frac{\alpha }{2}\sin2\theta \right] \end{array}$$

**Figure 1:**
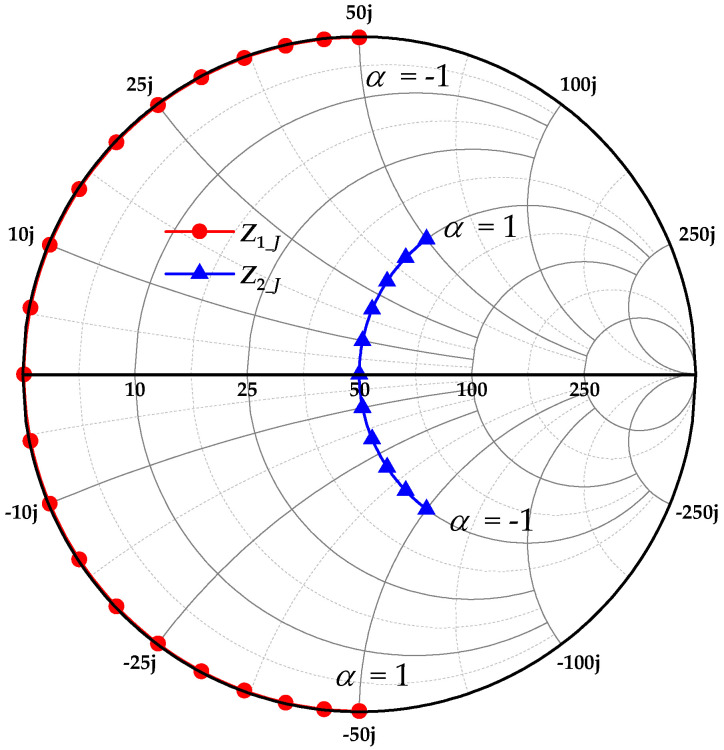
The normalized impedance traces of *Z_n_J_* with different values of *a*.

**Figure 2:**
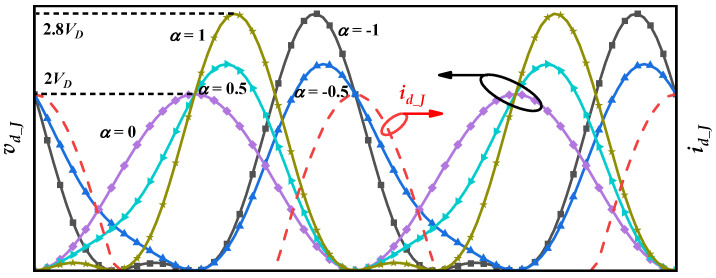
The waveforms of *v_d_J_* and *i_d_J_* for different values of *a*.

Here, α∈[−1, 1], *I_m_* and *V_D_* are the peak current and the bias voltage, respectively. The power amplifier operates in Class-J mode for all values of *a*, with the exception of *a* = 0 (Class-B). Based on Equations (1) and (2), the impedance conditions for continuous Class-J power amplifiers are

(3) $$Z_{n\_J}=\left\{\begin{array}{c} \begin{array}{c} \frac{2V_{D}}{I_{m}}\left(1+j\alpha \right),\;n=1 \end{array}\\ \begin{array}{c} -j\frac{3\pi V_{D}}{4I_{m}}\alpha ,\;n=2 \end{array}\\ \;\begin{array}{cc} 0,\; & n>2 \end{array} \end{array}\right.$$

where *n* is the harmonic order. Equation (3) demonstrates that different values of *a* produce distinct impedance traces on the Smith chart, as shown in Figure 1, which facilitates broadband matching of the amplifier. Furthermore, as the additional impedance introduced at the second-harmonic frequency is purely reactive, it theoretically imposes no degradation on the amplifier efficiency. Figure 2 shows the waveforms of *v_d_J_* and *i_d_J_* for different values of *a*. For the Class-J amplifiers, it can be observed that affected by the second harmonic, the waveform of *v_d_J_* shifts left or right under different values of *a* (relative to *a* = 0) [10].

## 3. Harmonic Load Pull

To achieve the design of a continuous-mode broadband high-efficiency power amplifier, in addition to performing fundamental load pull, it is also necessary to determine the range of the second harmonic impedance. The selected transistor based on a 0.25 mm GaAs pHEMT process features a maximum drain-source voltage of 18 V. As can be seen from Figure 2, in continuous-mode operation, the peak voltage of *v_ds_* exceeds the drain bias voltage *V_D_* by approximately 2.8 times. To avoid transistor breakdown caused by excessive peak voltage, *V_D_* is ultimately set to 6 V.

**Figure 3:**
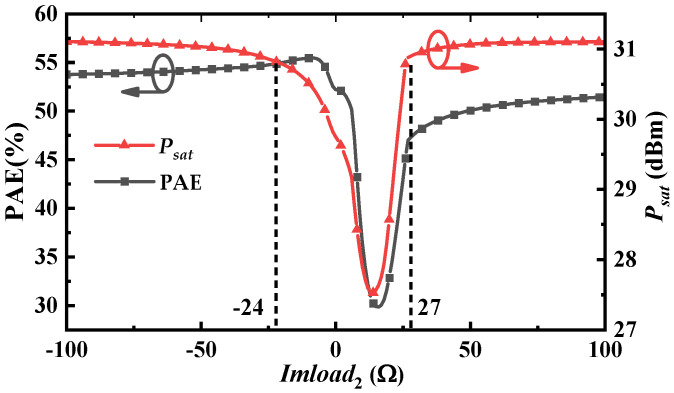
The simulated results of *P_sat_* and PAE at different values of *Imload*_2_.

Under a 6 V/−1 V bias condition, fundamental load pull is conducted on the output-stage transistors of the X-band and Ku-band power amplifiers. The resulting optimum impedances serve as the initial values for subsequent iterations. To simulate practical losses, the real part of the second harmonic impedance is fixed at 5 W, while a parameter sweep is performed over its imaginary part (*Imload*_2_). Figure 3 plots the saturated output power (*P_sat_*) and PAE of the X-band transistor versus *Imload*_2_. The results reveal a sharp degradation in both *P_sat_* and PAE within the *Imload*_2_ range of −*j*24 W to *j*27 W.

Based on the second harmonic parameter sweep, appropriate harmonic load values are selected. After multiple iterations of fundamental load-pull and harmonic impedance sweeps, the final fundamental and harmonic impedance values are obtained. Table 1 presents the results of source pull and harmonic load pull for the output-stage transistors of the X-band and Ku-band power amplifiers.

## 4. Design of the Harmonic Matching Network

**Figure 4:**
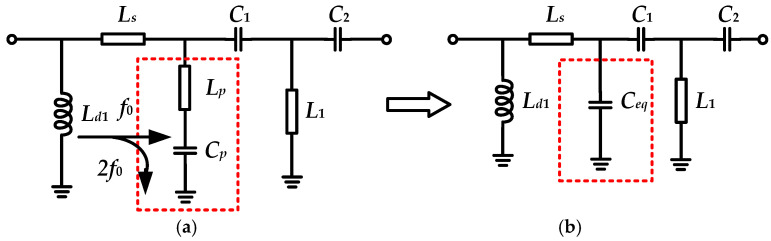
The schematic diagram of the (**a**) designed harmonic matching network and (**b**) its fundamental equivalent circuit.

As shown in Figure 1 and Table 1, the second harmonic load is purely reactive. Consequently, the output matching network must achieve a dual function: transforming the 50 Ω source impedance to the fundamental impedance *Z_f_*_0_, while concurrently providing the required harmonic impedance *Z*_2*f*0_. Although the off-chip branch-line structure can readily implement such harmonic matching, its large circuit area makes it unsuitable for integration in phased array T/R modules [11]. Fortunately, advances in semiconductor technology now permit the on-chip integration of harmonic control networks. While this approach may incur a slight efficiency penalty compared to off-chip, high-Q matching circuits, the significantly reduced amplifier area facilitates integration within T/R modules. Figure 4 shows the schematic diagram of the designed impedance matching network with a second harmonic control function.

In Figure 4, *L_p_* and *C_p_* resonate at 2*f*_0_ to realize second harmonic short-circuiting, whereas at the fundamental frequency, they are equivalent to a shunt grounded capacitor *C_eq_*. Equation (4) presents the relationship between *C_p_* and *C_eq_*. *L_s_* is employed to tune *Imload*_2_ to meet the requirements of *Z*_2*f*0_ specified in Table 1. *L*_1_, *C*_1_, *C*_2_, *C_eq_*, and *L_s_* form a fifth-order matching network, achieving fundamental impedance matching for both the X and Ku bands. The drain bias inductor *L_d_*_1_ is placed before the series resonant network; to avoid affecting the second harmonic, it has a large inductance value and does not participate in impedance matching.

(4) $$C_{p}=\frac{3}{4}C_{eq}$$

**Figure 5:**
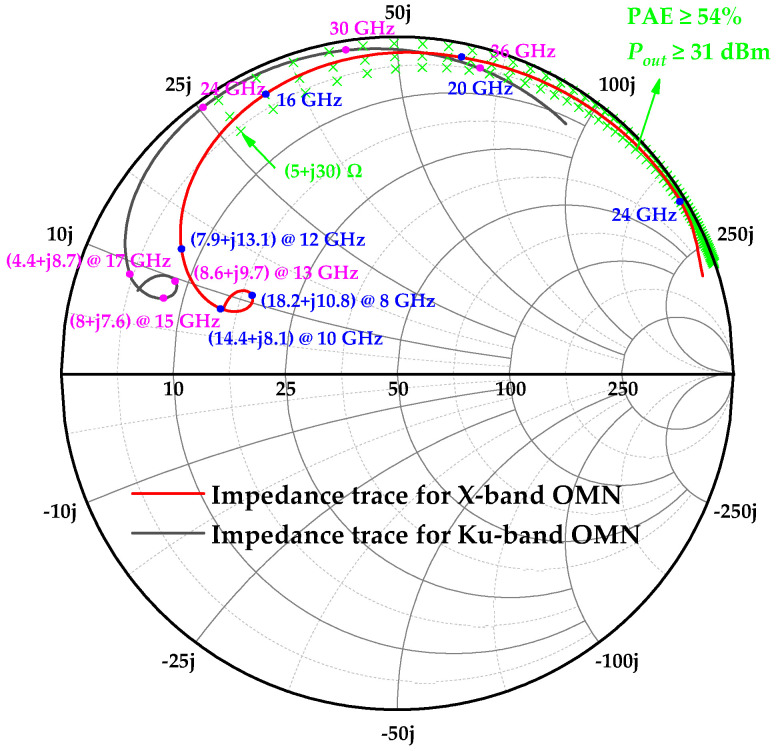
The simulated impedance traces of the OMNs for the X band and Ku band.

Figure 5 presents the simulated impedance traces of the output matching networks (OMNs) for the X band and Ku band. The highest efficiency and output power of the output-stage transistor are achieved when its second harmonic impedance is tuned to lie within the green region. Combined with Table 1, it can be seen that at both the frequency bands, the designed harmonic matching network can not only meet the second harmonic impedance control requirements but also satisfy the fundamental load matching requirements.

## 5. Design of the X and Ku Bands Class-J Power Amplifiers

**Figure 6:**
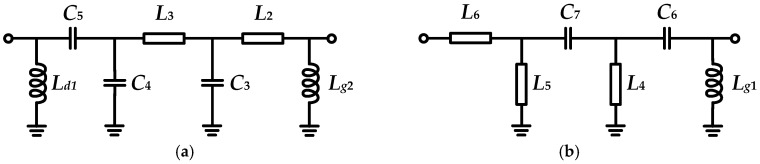
The schematic diagrams of the (**a**) inter-stage and (**b**) input matching networks.

After completing the output impedance matching, the driver stages of the X-band and Ku-band amplifiers are designed. Figure 6 presents the schematic diagrams of the interstage and input matching networks, where *L_d_* and *L_g_* serve as the drain and gate bias lines, respectively. Table 2 shows the source-pull and load-pull results of the driver transistor. Combined with *Z_s_* of the output transistors in Table 1, the component values are optimized.

**Figure 7:**
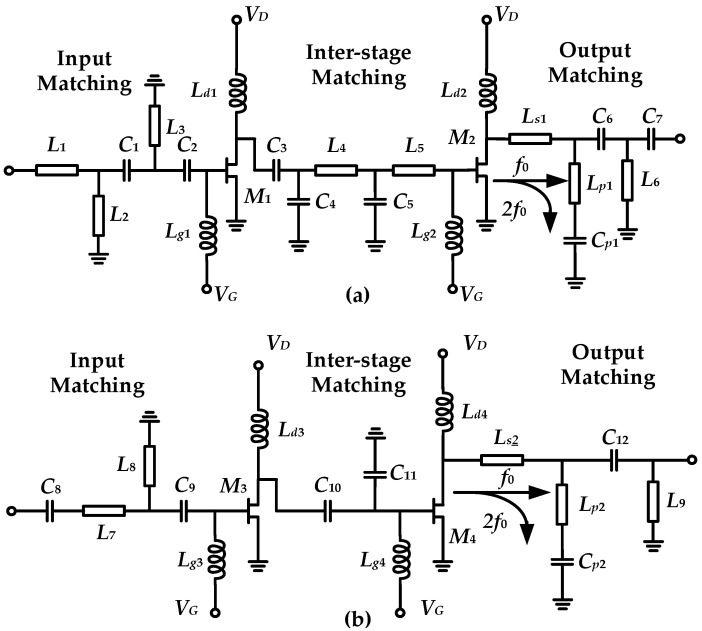
The schematic diagrams of the designed power amplifiers at (**a**) X band and (**b**) Ku band.

**Figure 8:**
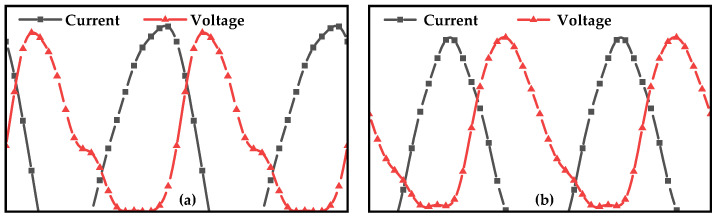
The output-stage voltage and current waveforms of the amplifiers operating at (**a**) 10 GHz and (**b**) 14 GHz.

Figure 7 shows the optimized schematic diagrams of the proposed X-band and Ku-band power amplifiers. The optimized component values are provided in Table 3. Among them, some components are omitted as their minimal contribution to matching. All inductors are implemented using microstrip lines. Figure 8 presents the output-stage drain-source voltage and current waveforms of the amplifiers operating in the X and Ku bands. It can be observed that affected by the second harmonic, the peaks of the drain voltage all shift to the left, which is consistent with the characteristics of Class-J power amplifiers.

## 6. Simulated and Measured Results

**Figure 9:**
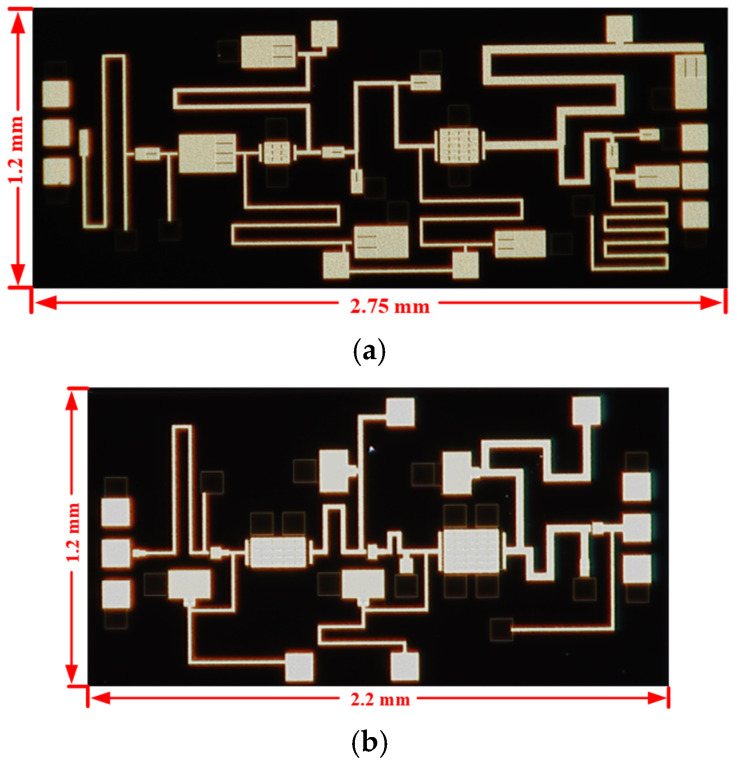
The microscope photograph of the designed continuous-mode power amplifiers for (**a**) the X band and (**b**) the Ku band.

**Figure 10:**
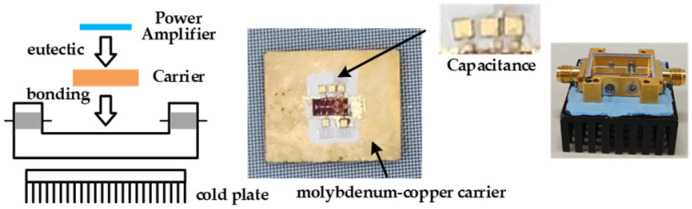
The mearsured carrier for the amplifier chips.

The two proposed continuous-mode power amplifiers are fabricated using a 0.25 mm GaAs pHEMT process. Figure 9 shows the microscope photographs of the two power amplifiers, with die areas of 2.75 mm × 1.2 mm and 2.2 mm × 1.2 mm, respectively. The S-parameters of the chips are measured using a Cascade Summit 1200 M probe station and a Keysight N5245A microwave network analyzer. Both chips undergo large-signal measurement after being eutectically bonded to a package with molybdenum-copper carriers, as shown in Figure 10. During the measurement, a heat sink is placed at the bottom to ensure efficient heat dissipation of the power amplifier chips.

**Figure 11:**
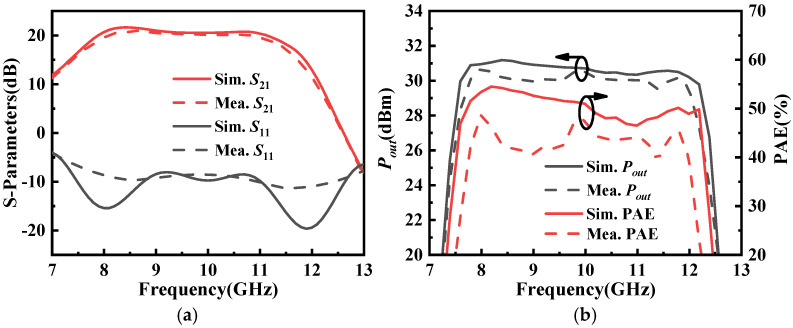
The simulated and measured results of the designed X-band power amplifier: (**a**) S-parameters and (**b**) large-signal characteristics.

Figure 11a presents the S-parameter simulated and measured results of proposed X-band power amplifier. It can be observed that the simulated results are in good agreement with the measured results. Within the frequency range of 8 GHz to 12 GHz, the power amplifier achieves an average small-signal gain of approximately 20 dB, and the measured input reflection coefficient is better than −8 dB. Figure 11b shows the large-signal characteristic simulated and measured results. Within the operating frequency band, the power amplifier delivers a saturated output power exceeding 30 dBm, with a PAE higher than 39% across the band and a maximum of 48.5%.

**Figure 12:**
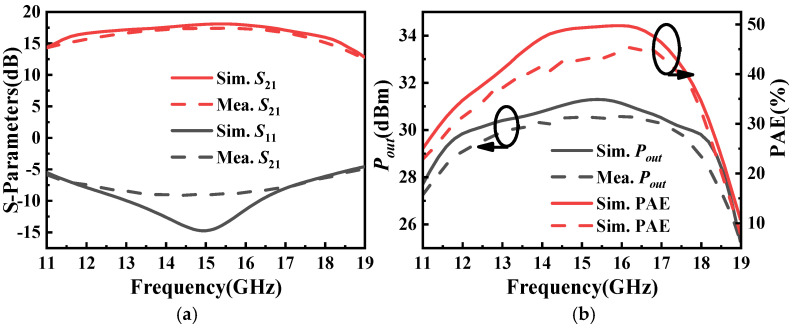
The simulated and measured results of the designed Ku-band power amplifier: (**a**) S-parameters and (**b**) large-signal characteristics.

The simulated and measured results of the designed Ku-band power amplifier are shown in Figure 12. Over the frequency range of 12 GHz to 18 GHz, the power amplifier achieves an average small-signal gain of approximately 16.5 dB, and the measured input reflection coefficient is better than −6.3 dB. The measured saturated output power achieves an average of 30.8 dBm across the band and 29 dBm at the band edges. Meanwhile, the measured PAE within the band reaches a maximum of 45.3% and an average of 40.32%.

The detailed performance metrics of two fabricated power amplifiers are listed in Table 4 and compared with several similar GaAs-process-based power amplifier studies in the literature [12,13,14,15,16,17]. Compared with the power amplifiers in [12,13,16], the proposed design demonstrates a higher PAE under a comparable bandwidth. Although [14,15] achieve high efficiency by employing only fundamental load-pull techniques, they are narrowband. The well-engineered matching network in [17] enables excellent bandwidth but sacrifices PAE.

**Table 4:** Comparison with other similar research on power amplifiers in GaAs process.

Ref.	Technology	Frequency (GHz)	BW (%)	*P_sat_* * (dBm)	PAE * (%)	Area (mm^2^)
[12]	0.15-μm GaAs	7~11.5	48.7	33.8	43	3.6 × 1.8
[13]	0.25-μm GaAs	8~13	47.6	31	35	1 × 3.5
[14]	0.25-μm GaAs	8.5~11.5	30	35	49	3 × 1.8
[15]	0.25-μm GaAs	8.5~9.5	11.1	29.6	44.4	2 × 2
		15.5~16.5	6.3	30.1	38.4	
[16]	0.25-μm GaAs	11.7~17.6	40.3	31	38.8	3.8 × 3.3
[17]	0.25-μm GaAs	6~18	100	40.5	29	5 × 3.6
This work	0.25-μm GaAs	8~12	40	31.2	48	2.75 × 1.2
		12~18	40	30.8	45.3	2.2 × 1.2

* maximum values.

## 7. Conclusions

In this article, two wideband high-efficiency power amplifiers operating in the X and Ku bands, respectively, are designed and fabricated using a 0.25 µm-GaAs pHEMT process. A harmonic matching network is introduced in both power amplifiers, which extends the operating bandwidth without sacrificing efficiency. The proposed X-band power amplifier achieves a peak saturated output power of 31.2 dBm and a maximum PAE of 48%. The proposed Ku-band amplifier delivers a peak saturated output power of 30.8 dBm and a maximum PAE of 45.3%. Both chips feature a compact footprint, rendering them suitable for integration into miniaturized phased array T/R modules.

## Figures and Tables

**Table 1:** The results of source pull and harmonic load pull for the output-stage transistors of the X-band and Ku-band power amplifiers.

Frequency	8 GHz	10 GHz	12 GHz
*Z_s_*	(11.9 + *j*21.5) Ω	(5.2 + *j*15) Ω	(5.8 + *j*14.3) Ω
*Z_f_*_0_	(18.5 + *j*11.6) Ω	(14.6 + *j*9.1) Ω	(10.1 + *j*10.4) Ω
*Z*_2*f*0_	>*j*30 W	>*j*30 W	>*j*30 W
**Frequency**	**13 GHz**	**15 GHz**	**17 GHz**
*Z_s_*	(7.3 + *j*10.8) Ω	(6.3 + *j*2.4) Ω	(3.1 + *j*1.7) Ω
*Z_f_*_0_	(8.9 + *j*9.5) Ω	(7.8 + *j*8.1) Ω	(6.1 + *j*8.5) Ω
*Z*_2*f*0_	>*j*30 W	>*j*30 W	>*j*30 W

**Table 2:** The results of source pull and load pull for the driver transistors of the X band and Ku band.

Frequency	8 GHz	10 GHz	12 GHz
*Z_s_*	(17.4 + *j*16) Ω	(15.2 + *j*14.5) Ω	(13.9 + *j*12.1) Ω
*Z_L_*	(31.3 + *j*28.5) Ω	(29.5 + *j*26) Ω	(28.6 + *j*23) Ω
**Frequency**	**13 GHz**	**17 GHz**	**15 GHz**
*Z_s_*	(2.4 + *j*4.6) Ω	(2 + *j*5.2) Ω	(2 + *j*4.3) Ω
*Z_L_*	(9.3 + *j*14.6) Ω	(7.1 + *j*12) Ω	(3.9 + *j*13) Ω

**Table 3:** The optimized component values shown in Figure 6.

Components	Values	Components	Values
*L*_1_	10 mm × 1500 μm	*C*_12_	0.52 pF
*L*_2_	10 mm × 340 μm	*L_g_*_1_	10 mm × 1550 μm
*L*_3_	10 mm × 300 μm	*L_g_*_2_	10 mm × 1400 μm
*L*_4_	15 mm × 400 μm	*L_g_*_3_	10 mm × 900 μm
*L*_5_	15 mm × 300 μm	*L_g_*_4_	10 mm × 1000 μm
*L*_6_	10 mm × 1800 μm	*L_d_*_1_	15 mm × 1350 μm
*L*_7_	10 mm × 1050 μm	*L_d_*_2_	30 mm × 1600 μm
*L*_8_	10 mm × 250 μm	*L_d_*_3_	15 mm × 650 μm
*L*_9_	10 mm × 800 μm	*L_d_*_4_	30 mm × 1100 μm
*C*_1_	0.67 pF	*L_s_*_1_	20 mm × 450 μm
*C*_2_	6.10 pF	*L_s_*_2_	30 mm × 400 μm
*C*_3_	0.52 pF	*L_p_*_1_	10 mm × 160 μm
*C*_4_	0.62 pF	*L_p_*_2_	30 mm × 120 μm
*C*_5_	1.01 pF	*C_p_*_1_	0.39 pF
*C*_6_	0.68 pF	*C_p_*_2_	0.35 pF
*C*_7_	2.30 pF	*M*_1_	6 × 100 μm
*C*_8_	0.19 pF	*M*_2_	10 × 150 μm
*C*_9_	0.52 pF	*M*_3_	8 × 200 μm
*C*_10_	0.56 pF	*M*_4_	12 × 200 μm
*C*_11_	0.83 pF		

## Data Availability

The data presented in this study are available on request from the corresponding author due to (specify the reason for the restriction).

## References

[1] Maini A.K. (2018). Handbook of Defence Electronics and Optronics: Fundamentals, Technologies and System.

[2] Batra J., Balakrishnan G., Aiello R., Foerster J.R., Dabak A. (2004). Design of a multiband OFDM system for realistic UWB channel environments. IEEE Trans. Microw. Theory Techn..

[3] Cripps S.C., Tasker P.J., Clarke A.L., Lees J., Benedikt J. (2009). On the continuity of high efficiency modes in linear RF power amplifiers. IEEE Microw. Wirel. Compon. Lett..

[4] Chu C., Sharma T., Dhar S.K., Darraji R., Wang X., Pang J., Zhu A. (2021). Waveform engineered sequential load modulated balanced amplifier with continuous class-F^−^ 1 and class-J operation. IEEE Trans. Microw. Theory Tech..

[5] Xuan X., Cheng Z., Zhang Z., Le C. (2023). Highly efficient ultrawideband power amplifier based on a novel multi-branch matching network. IEEE Microw. Wirel. Technol. Lett..

[6] Alizadeh A., Hassanzadehyamchi S., Medi A., Kiaei S. (2020). An X-band class-J power amplifier with active load modulation to boost drain efficiency. IEEE Trans. Circuits Syst. I Regul. Pap..

[7] Gong T., Cheng Z., Zheng B., Xuan X., Le C., Fan W., Zhang Z. (2024). SC band wideband GaN power amplifier MMIC for radar application. IEICE Electron. Express.

[8] Wright P., Lees J., Tasker P.J., Benedikt J., Cripps S.C. (2009). An Efficient, Linear, Broadband Class-J-Mode PA Realised Using RF Waveform Engineering. Proceedings of the 2009 IEEE MTT-S International Microwave Symposium Digest.

[9] Alizadeh A., Hassanzadehyamchi S., Medi A. (2019). Integrated output matching networks for class–J/J− 1 power amplifiers. IEEE Trans. Circuits Syst. I Regul. Pap..

[10] Kazimierczuk M.K. (2014). RF Power Amplifiers.

[11] Sheikhi A., Hemesi H. (2021). Analysis and design of the novel class-F/E power amplifier with series output filter. IEEE Trans. Circuits Syst. II Express Briefs.

[12] Li J., Yuan Y., Yuan B., Tan C., Yu Z. (2024). Analysis and design of broadband 2-W power amplifier based on cascode transistors. Microw. Opt. Technol. Lett..

[13] Babakrpur E., Medi A., Namgoong W. (2017). Wideband GaAs MMIC driver power amplifiers for X and Ku bands. Proceedings of the 2017 Texas Symposium on Wireless and Microwave Circuits and Systems (WMCS).

[14] Hua Y., Wu H., Liao X., Liao C., Hu L., Lv J. (2018). A High-Efficiency 3-Watt GaAs pHEMT X-Band MMIC Power Amplifier. Proceedings of the 2018 International Conference on Microwave and Millimeter Wave Technology.

[15] Xie C., Wu P., Tan C., Yuan Y., Zeng J., Yu Z. (2022). An X/Ku dual-band switchless frequency reconfigurable GaAs power amplifier. IEEE Microw. Wirel. Compon. Lett..

[16] Yao G., Jia H., Zhao Z., Lu Y., Yi C., Liu X., Feng T., Yang L.A., Ma X. (2024). A Ku-Band Broadband High-Efficiency GaAs MMIC Power Amplifier. Proceedings of the 2024 International Conference on Microwave and Millimeter Wave Technology (ICMMT).

[17] Meghdadi M., Medi A. (2017). Design of 6–18-GHz high-power amplifier in GaAs pHEMT technology. IEEE Trans. Microw. Theory Tech..

